# Mechanochemical Effect on Controlled Drug Release of Konjac Glucomannan Matrix Tablets during Dry Grinding

**DOI:** 10.3390/gels8030181

**Published:** 2022-03-15

**Authors:** Fuminori Okazaki, Yusuke Hattori, Tetsuo Sasaki, Makoto Otsuka

**Affiliations:** 1 Faculty of Pharmacy, Musashino University, 1-1-20 Shinmachi, Nishi-Tokyo 202-8585, Tokyo, Japan; s1343048@stu.musashino-u.ac.jp (F.O.); yhattori@musashino-u.ac.jp (Y.H.); 2 Graduate School of Medical Photonics, Shizuoka University, 3-5-1 Johoku, Naka-ku, Hamamatsu 432-8011, Shizuoka, Japan; sasaki.tetsuo@shizuoka.ac.jp; 3 Research Institute of Electronics, Shizuoka University, 3-5-1 Johoku, Naka-ku, Hamamatsu 432-8011, Shizuoka, Japan

**Keywords:** konjac glucomannan, solid-state mechanochemical reaction, swelling and disintegration property, Fourier-transform infrared spectral change, polymer matrix tablets, sustained drug release kinetics

## Abstract

To design a controlled drug release preparation based on a safe natural material, a Konjac glucomannan (KGM) mixture containing 16.0 *w/w*% calcium hydroxide (Ca(OH)_2_) was ground in a planetary ball mill for 0–120 min. The mechanochemical effect on the physicochemical properties of the KGM ground product was investigated by Fourier-transform infrared spectroscopy (FT-IR), powder X-ray spectroscopy, scanning electron microscopy with energy-dispersive X-ray spectroscopy, and drug release testing. The FT-IR spectra of the ground KGM indicated that the deacetylation reaction of KGM was accelerated in the Ca(OH)_2_-containing sols by mechanochemical energy, and the degree of deacetylation of KGM was dependent on the grinding time. The time required for tablet disintegration of the KGM matrix tablets containing theophylline increased as the grinding time increased; therefore, drug release was sustained. The Higuchi plots of the matrix tablets obtained from KGM ground for 60–120 min exhibited good linearity because they maintained their gel matrix tablet shape during the release test. However, KGM tablets ground for 0–30 min exhibited nonlinear curves, which were caused by tablet disintegration. This suggests that drug release from the KGM matrix tablet can be freely controlled by the degree of mechanochemical treatment.

## 1. Introduction

Sustained-release tablets assist in dosing compliance [1] and improve patient quality of life by reducing dosing frequency. Various types of synthetic hydrophilic polymers that can form a gel matrix are used as bases for sustained-release tablets [2]. Since hydrated gel layers form on the surface of the gel matrix tablets, the dissolution and erosion rates of the gel layer depend on the properties and contents of the polymer excipients used, and drug release can be controlled for 8–24 h [3,4,5,6].

Naturally abundant polysaccharides have attracted considerable attention as a base for sustained-release preparations because they are non-toxic, safe, hydrophilic, and biodegradable. Glucomannan (GM) is a non-ionic polysaccharide extracted from the tubers of Konjac, which is a high-molecular-weight compound that has also been used in food [7,8]. Konjac glucomannan (KGM) fine powder obtained from natural products is partially acetylated GM, which can be gelatinized by heating in an alkaline solution, followed by deacetylation [9]. According to the FDA, GM is recognized as a safe biomedical material and is used worldwide as a functional healthcare material for diabetes and steatosis [10]. KGM is not hydrolyzed by salivary and pancreatic amylase owing to the presence of the 1,4 bonds of β-D-mannose and glucose units; therefore, KGM can enter the colon as ingested and is fermented by colonic bacteria [10]. KGM is one of the most viscous dietary fibers because it is water-soluble and highly absorbent. KGM forms a strong, elastic, and heat-stable gel matrix when heated in a weakly alkaline solution. Because of the effective properties of KGM gel powder (such as hydrophilicity, thickening, stability, and the ability to form emulsions, suspensions, gels, and films [9]), KGM can control drug release and has great potential as a pharmaceutical additive. Therefore, KGM was evaluated for its ability to control drug release in gel matrix tablets containing various drugs, and its application as a drug delivery system was investigated [11,12]. The gel matrix tablets were compression-prepared via a conventional wet granulation process [13,14,15]. However, since the controlled-release function of these matrix tablets depended on the amount of the prescribed ingredient, it was difficult to adjust and change the release rates of the tablets because the prescription of the formulations had to be changed. Studies were conducted on the development of pharmaceutical additives with a controlled drug release function [16,17]. Therefore, to utilize natural resources as safe medical additives without complicated process operations, this study focused on mechanochemical treatment technology, which controls the crystallinity of natural product-derived additives. Mechanochemical reactions allow the synthesis of compounds, phases, and microstructures that are essentially different from those obtained by conventional chemical reactions [18]. Using the mechanochemical reactions of organic compounds generated by grinding energy, drugs can be bonded with biopolymers [19], amorphous solids can be formed to improve the solubility of drugs with poor water solubility [20], and drugs can form co-crystals with specific compounds to become chemically stable [21,22]. In previous studies [17,23], the amorphization of starch and its gel formation in solution were controlled by ball mill grinding, and the drug release rates from the gel matrix tablets were controlled using drug release kinetic tests and X-ray computed tomography imaging.

Therefore, in this study, to control drug release from the device by a simple manufacturing process and avoid the formation of a KGM complex with other polymer compounds in wet granulation, as previously reported [11,12,13,14,15], KGM and alkaline substances were mechanochemically treated in a dry-state-grinding process using a planetary ball mill. The KGM crystallinity and the dispersion uniformity of alkaline fine particles depend on their mechanochemical energy. The ground KGM preparations obtained by mechanochemical reactions and their compressed tablets were examined for their chemical and pharmaceutical properties related to controlling drug release.

## 2. Results

### 2.1. Change in the Physicochemical Properties of the Ground KGM and Ca(OH)_2_ Mixed Powder

**Figure 1:**
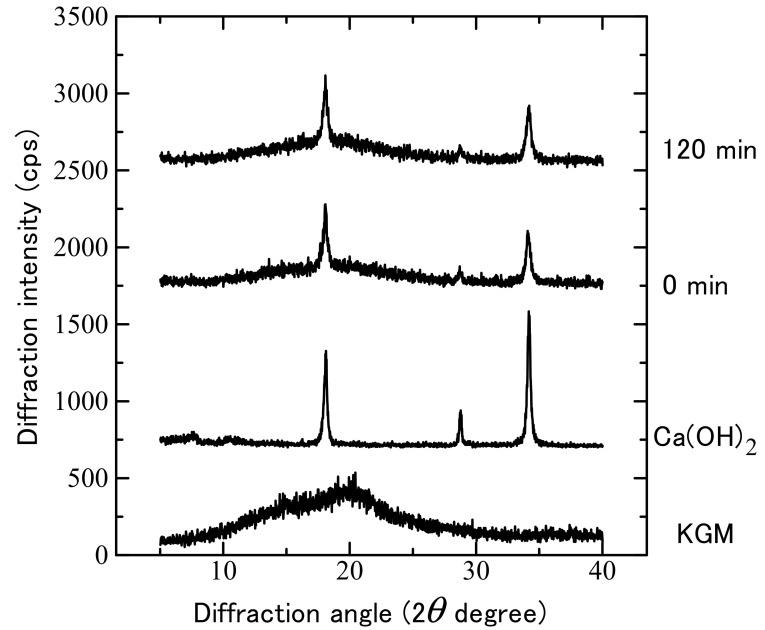
Powder X-ray diffraction profiles of the KGM mixtures containing 16.0 *w/w*% Ca(OH)_2_ after various grinding times.

Figure 1 shows the XRD profiles of the KGMH mixtures containing 16 *w/w*% Ca(OH)_2_ after various grinding times. The XRD profile of Ca(OH)_2_ showed specific diffraction peaks at 18°, 29°, and 34°, whereas the profile of KGM showed a halo pattern without any diffraction peaks. Therefore, the profile of KGMH0 (a physical mixture) only showed specific peaks due to Ca(OH)_2_, and the profiles of the ground KGMH mixtures (KGMH120) did not change significantly up to 120 min of grinding.

**Figure 2:**
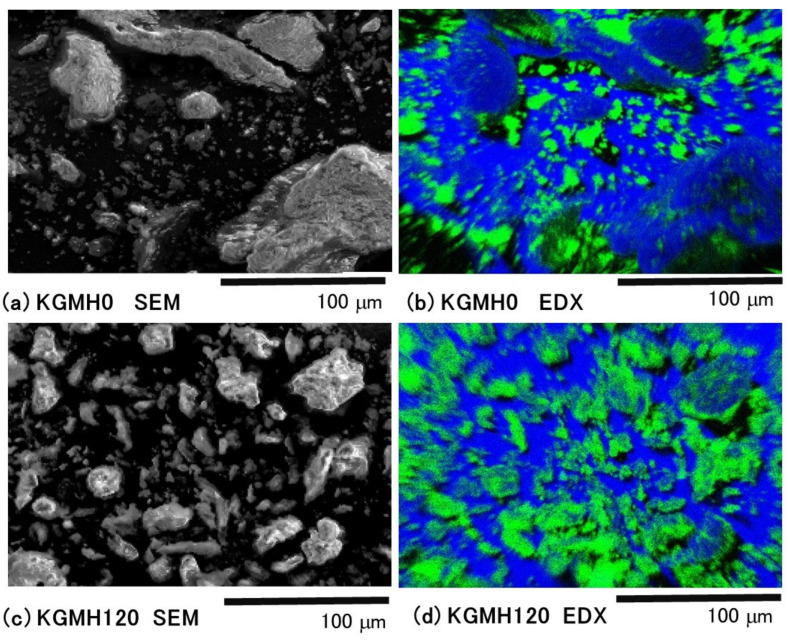
Scanning electron microscopy/energy-dispersive X-ray spectroscopy images of the KGMH0 and KGMH120 particles, respectively. SEM, scanning electron microscopy (**a**,**c**); EDX, energy-dispersive X-ray spectroscopy Ca (green) in EDX (**b**,**d**).

Figure 2 shows SEM/EDX images of the KGMH0 and KGMH120 powders. In the SEM image of KGMH0, many particles (5–150 μm in diameter) were observed, and they had a wide particle size distribution. However, only smaller particles (5–20 μm in diameter) were observed in the EDX image. In contrast, in the SEM image of KGMH120, a large number of particles (5–40 μm in diameter) were observed, and the EDX and SEM images of the particles were almost identical.

**Figure 3:**
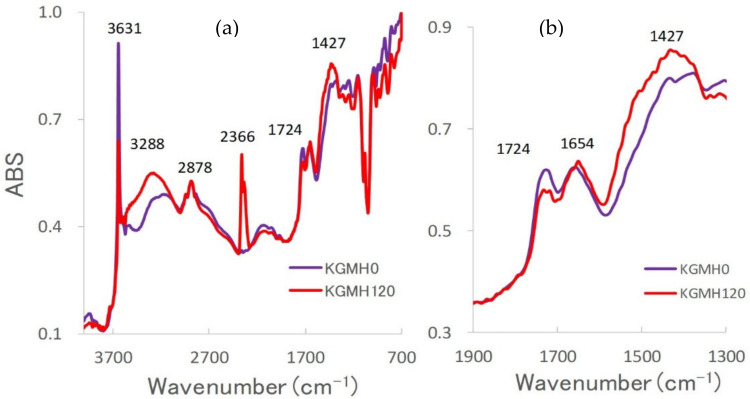
Fourier-transform infrared spectra of the KGMH mixtures ground for various grinding times. (**a**) Spectral range: 4000–700 cm^−1^; (**b**) spectral range: 1900–1300 cm^−1^.

Figure 3 shows FT-IR spectra of the KGMH mixtures after various grinding times. The IR spectra of the ground KGMH mixtures exhibited absorption peaks at 3631, 3288, 2878, 2366, 1724, 1654, and 1427 cm^−1^. The absorption intensity of the peaks at 3621 and 1724 cm^−1^ decreased with increasing grinding time. In contrast, the absorption intensity of the peaks at 3288, 2366, and 1427 cm^−1^ increased with increasing grinding time. These results suggest that the molecular interaction of KGM changed significantly after grinding.

### 2.2. Effect of Grinding on the Drug Release Profiles of the KGM Matrix Tablets

**Figure 4:**
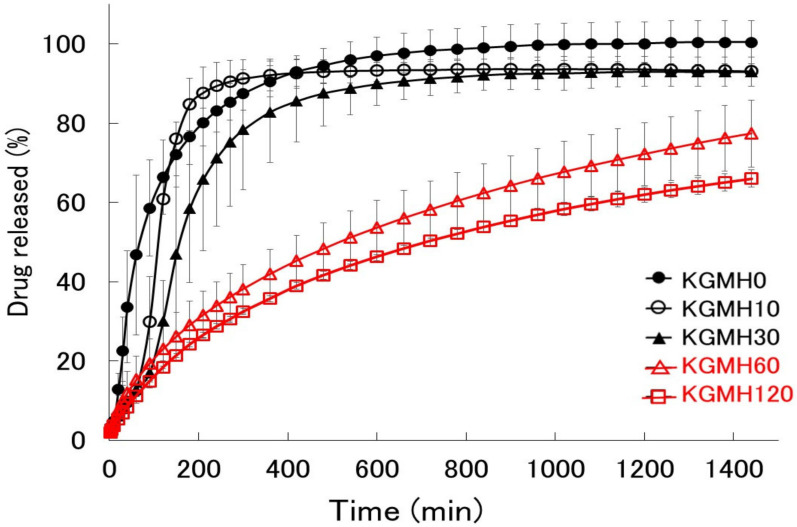
Drug release profiles of the polymer matrix tablets prepared from the KGMH mixtures ground for 0–120 min.

Figure 4 shows the drug release profiles of the polymer matrix tablets prepared from KGMH mixtures ground for 0–120 min. The KGMH0 tablets disintegrated at approximately 30 min and released over 75% of the drug within 150 min. However, based on the drug release profiles of ground KGMH tablets, the time required for tablet disintegration increased as the grinding time increased, and drug release was suppressed. Drug release from the KGMH tablets was delayed with increasing grinding time because a typical gel layer was visibly formed on the outside of the KGMH60 and KGMH120 tablets.

**Figure 5:**
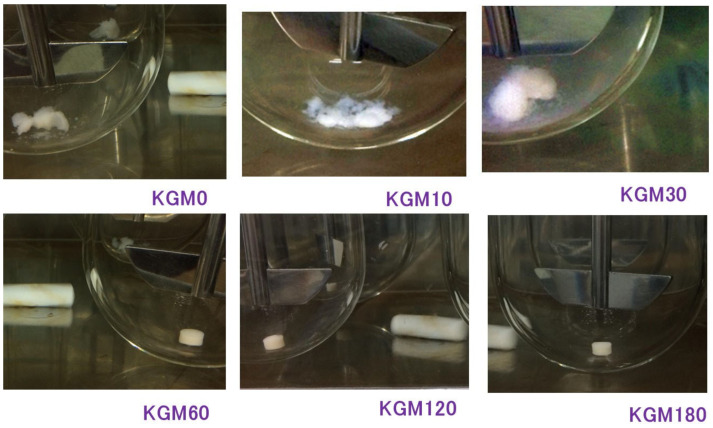
Photographs of the KGMH tablets after 24 h during the drug release tests.

Figure 5 shows photographs of the KGMH tablets after 24 h during the drug release tests. After 24 h, the KGMH0, KGMH10, and KGMH30 tablets had completely disintegrated, but the KGMH60 and KGMH120 tablets retained a tablet shape. This result indicates that it was possible to control drug release of the KGMH polymeric matrix tablets by mechanochemical treatment in a ball mill. Therefore, it was hypothesized that the drug release profiles from the KGMH60 and KGMH120 matrix tablets followed the Higuchi Equation (1) [24]:

(1) $$Q=\sqrt{\frac{D\varepsilon }{\tau }\left(2A-\varepsilon C_{s}\right)C_{s}t}=K_{H}t^{\frac{1}{2}}$$

where Q is the amount of drug released per unit surface area, *A* is the drug amount per unit volume in the device, *D* is the diffusion coefficient, *ε* is the device porosity, *τ* is the tortuosity, *C_s_* is the drug solubility, and *K_H_* is the Higuchi drug release rate constant.

**Figure 6:**
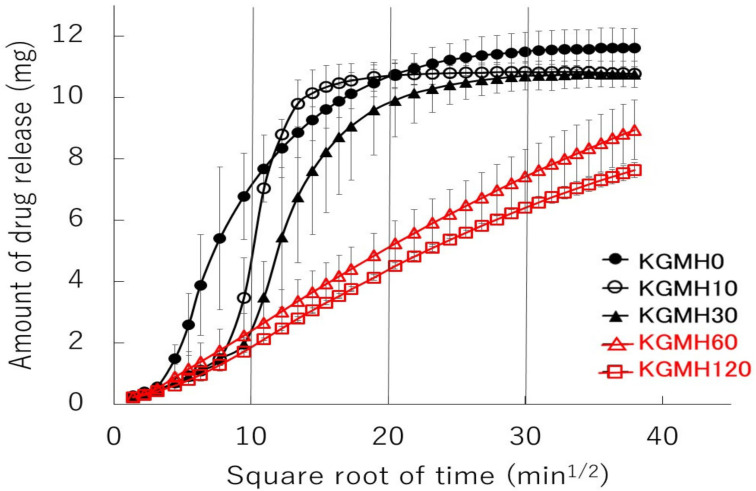
Higuchi-type plots of the amount of drug released from the KGMH matrix tablets against the square root of time.

**Figure 7:**
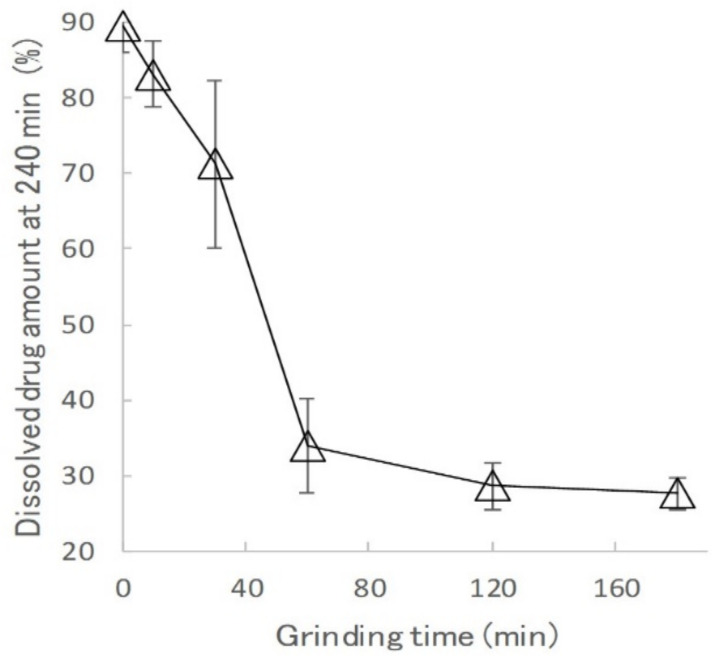
Effect of grinding time on the drug release amount (RA4) of the KGMH matrix tablets after 240 min dissolution test.

Figure 6 shows Higuchi-type plots of the amount of drug released from the KGMH matrix tablets against the square root of time. The plots of the matrix tablets composed of KGMH ground for 60 min or longer exhibited good linearity (coefficient of determination above 0.99) and followed the Higuchi Equation (1). In contrast, the curves of the KGMH tablets ground for 30 min or less were nonlinear owing to tablet disintegration and did not follow the same drug release mechanism. Therefore, the drug release rate constants (K_H_) of the KGMH60 and KGMH120 matrix tablets, which were calculated based on Equation (1) using the least-squares method, were 0.246 and 0.215 mg/min^1/2^, respectively. Since the Higuchi release theory could not be applied to all tablet samples (as shown in Figure 6), a different method was applied to quantitatively evaluate the amount of drug released in a limited time. Figure 7 shows the effect of grinding time on the drug release amount (RA4) of the KGMH matrix tablets after the 240 min dissolution test. The RA4 values of the KGMH matrix tablets decreased significantly with increasing grinding time, and the RA4 values of the KGMH0, KGMH10, and KGMH30 matrix tablets were significantly higher than those of the KGMH60 and KGMH120 matrix tablets.

**Figure 8:**
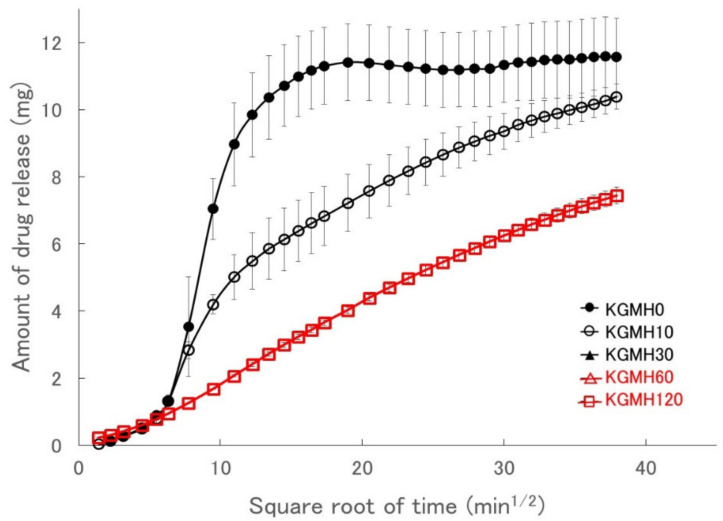
Effect of calcium hydroxide content on the drug release profiles of the KGML120, KGMM120, and KGMH120 matrix tablets.

To investigate the effect of the amount of Ca(OH)_2_, the KGM mixtures containing 5.2 and 10.5 *w/w*% Ca(OH)_2_ were ground for 120 min, respectively, and labeled KGML120 and KGMM120. Figure 8 shows the effect of Ca(OH)_2_ on the drug release profiles of the KGML120, KGMM120, and KGMH120 matrix tablets. While the Higuchi plot of the KGMH120 matrix tablets was a straight line, the plots of the KGMM120 and KGML120 matrix tablets were nonlinear curves, and their drug release did not follow the Higuchi theory. The release profiles of the matrix tablets were suppressed, reflecting the visually observed delayed tablet disintegration times.

**Figure 9:**
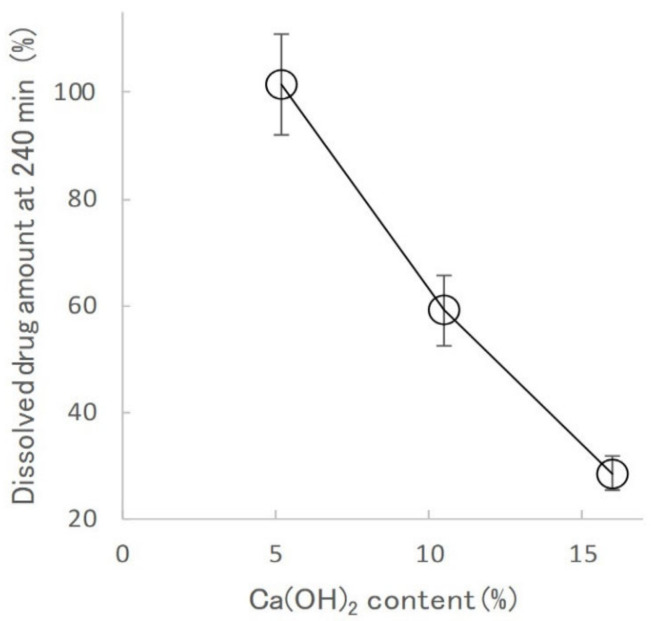
Effect of calcium hydroxide content on the RA4 of the KGM matrix tablets.

Figure 9 shows the effect of Ca(OH)_2_ on the RA4 value of KGM matrix tablets. The RA4 value of KGMH matrix tablets decreased significantly with increasing amounts of Ca(OH)_2_, with values of 101.5%, 59.1%, and 28.6% for KGML120, KGMH120, and KGMH120, respectively.

**Figure 10:**
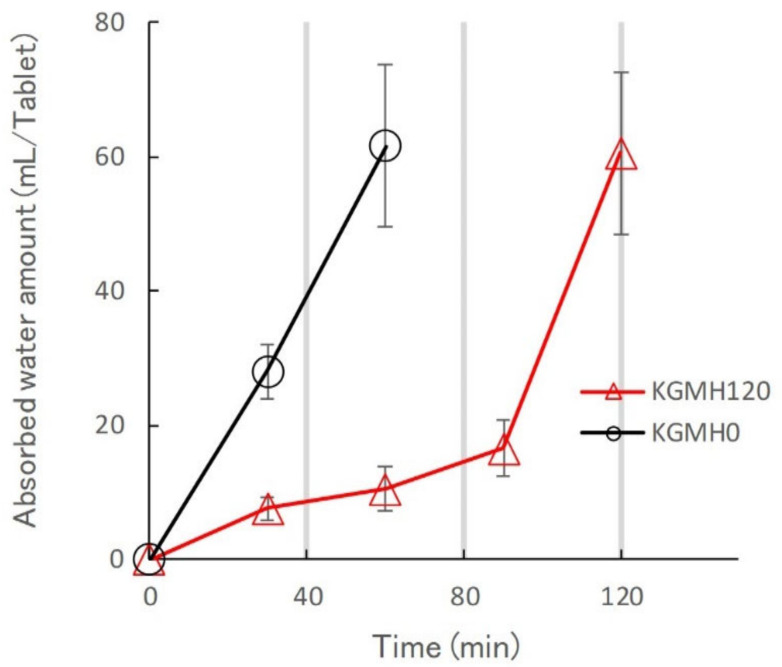
Effect of grinding on the water penetration rate of the KGMH0 and KGMH120 matrix tablets.

Figure 10 shows the effect of grinding on the water penetration rate of KGMH0 and KGMH120 matrix tablets. The KGMH0 tablets immediately absorbed water at the start of the release test. In contrast, the KGMH120 tablet slowly absorbed water, forming a gel layer on the surface at the beginning of the release test, but rapidly absorbed water after 100 min.

## 3. Discussion

### 3.1. Mechanochemical Reaction of KGM with Ca(OH)_2_ during Grinding

Since the KGM fine powder is a natural product, it cannot form a gel in water owing to partial acetylation of the GM. However, when KGM is partially deacetylated by heating in an alkaline solution, it can form a strong gel in water, depending on the degree of deacetylation, as previously reported [10]. When KGM is used as a base for sustained-release preparation for a drug delivery system, gelatinization treatment by heating in an alkaline solution is required, in which the chemical stability of the active pharmaceutical ingredients can be compromised. Therefore, in this study, we aimed to develop a dry solid-state gelatinization treatment for KGM that does not decompose active pharmaceutical ingredients.

After many trial studies, we found that KGM formulations with 5–16 *w/w*% Ca(OH)_2_ under dry grinding conditions were optimal for gelatinized matrix tablets for sustained drug release. The XRD profiles of the KGMH mixtures before and after the mechanochemical treatment showed a halo pattern with additional diffraction peaks due to Ca(OH)_2_, indicating that the XRD profile of KGMH120 was not significantly different before and after the treatment (Figure 1). The results suggested that most of the Ca(OH)_2_ did not change its basic crystal structure during the grinding process under the experimental conditions. In addition, the morphologies of the KGMH120 and KGMH0 particles observed by SEM/EDX (Figure 2) indicated that fine Ca(OH)_2_ particles (less than a few micrometers in diameter) were uniformly dispersed in the KGMH120 composite particles (5–40 μm in diameter) after mechanochemical treatment.

Furthermore, in the FT-IR spectra, the peaks of the ground KGMH mixtures (Figure 3) were assigned based on a previous study. The peaks at 3631 and 3288 cm^−1^ were attributed to the free and hydrogen-bonded stretching vibrations of the OH group [25]. The peaks at 2878 and 1427 cm^−1^ were due to the stretching and angular vibrations of CH in the alkene [25] and CO_3_^2-^ of CaCO_3_ [26], respectively. The peak at 1724 cm^−1^ was attributed to the stretching vibration of C=O in the esters’ acetyl groups [27]. The peaks at 2366 and 1654 cm^−1^ were attributed to the CO stretching vibration in adsorbed carbon dioxide gas [28] and the stretching vibration of OH in water [25], respectively.

The mechanochemical reaction between KGM and Ca(OH)_2_ can be explained using the following Equation (2):

(2) $$\begin{array}{c} \;\mathrm{KGM}+n\mathrm{Ca}{\left(\mathrm{OH}\right)}_{2}+{nH}_{2}O+{n\mathrm{CO}}_{2}\;\\ \;\;\;\;\;\;l\\ \;\;\;{\left({\mathrm{CH}}_{2}{\mathrm{OCOCH}}_{3}\right)}_{2n}\\ \;\;\;∆E\\ \;======>\;\;\;\;\;\;\;\;\mathrm{KGM}+2{n\mathrm{CH}}_{3}\mathrm{COOH}+n\mathrm{Ca}{\mathrm{CO}}_{3}\\ \;\;\;\;\;\;\;\;\;\;\;l\\ \;\;\;\;\;\;\;\;{\left({\mathrm{CH}}_{2}\mathrm{OH}\right)}_{2n} \end{array}$$

where *ΔE* is mechanochemical energy during grinding.

First, when Ca(OH)_2_ particles were ground in a ball mill, the reduction in particle size increased the surface area by more than 100 times (Figure 2c); therefore, carbon dioxide gas was adsorbed on the surface from the air in the environment (as shown by the increasing intensity of the absorbance peak at 2366 cm^−1^ in Figure 3). Fine Ca(OH)_2_ particles were uniformly distributed on KGM (Figure 2d), which may have formed alkaline sols (containing OH- ions) on the surface of the KGM powder with a small amount of water (peak at 1654 cm^−1^ in Figure 3). Second, the KGM deacetylation reaction was catalyzed and accelerated by Ca(OH)_2_-containing sols, as shown by the decrease in the peak at 1724 cm^−1^ due to solid-state grinding. Ca^2+^ ions reacted with carbon dioxide to produce CaCO_3_ (as shown by the increasing intensity of the absorbance peak at 1427 cm^−1^ in Figure 3). Third, when the grinding time for the partially deacetylated KGM [9] was increased, the absorbance of the peak at 3621 cm^−1^ decreased while the absorbance of the peak at 3288 cm^−1^ increased (Figure 3). This indicates that free OH groups were easily transformed into intermolecular hydrogen bond OH groups by mechanochemical energy. This phenomenon indicates that the steric hindrance caused by the KGM acetyl groups was reduced during partial deacetylation by the mechanochemical reaction. On the other hand, the solid-state intermolecular hydrogen bonds between the particle surfaces of the partially deacetylated KGM increased. Therefore, the ground KGMH mixtures facilitated the formation of a strong gel in an aqueous solution and suppressed tablet disintegration.

### 3.2. Effect of Grinding on the Drug Release Kinetics of KGM Matrix Tablets and Hydrogel Formation Mechanism

To optimize the pharmaceutical formulation of KGM as a sustained-release drug preparation, the effect of Ca(OH)_2_ content on the drug release profile of the KGM matrix tablets was investigated. When KGM was heat-treated with 5 *w/w*% Ca(OH)_2_ in a solution at over 90 °C, it formed a sufficiently strong gel [29,30]. Therefore, the standard formulation of KGM for gel formation was labeled as KGML, which contained approximately 5 *w/w*% Ca(OH)_2_ [29]. KGML120 and KGMM120 tablets obtained after grinding for 120 min began to disintegrate after approximately 30 min, as shown in the drug release profile (Figure 8). However, because the KGMH120 tablet containing 16 *w/w*% Ca(OH)_2_ formed a strong gel during the drug release test, the dissolution kinetics (Figure 8) were based on the Higuchi drug release theory [24]. Therefore, using the dry grinding method, 16 *w/w*% Ca(OH)_2_ was required to obtain a sustained-release drug preparation that followed Higuchi’s theory. To clarify the intermolecular formation mechanism of the KGM matrix tablets during the drug release test, the relationship between the grinding time and sustained drug release kinetics of the KGM matrix tablets was investigated. The drug release profiles of the KGMH matrix tablets showed that the drug release was delayed (Figure 6) as the mechanochemical reaction progressed (shown in the FT-IR spectra in Figure 3) with increasing grinding time.

As described in the previous section, KGM reacted with Ca(OH)_2_ sol in the solid-state through mechanochemical energy, and the KGM acetyl groups partially decomposed with increasing grinding time (Figure 3). Partially deacetylated KGM obtained by mechanical treatment can form a strong hydrogel [31]. After tablet compression, the deacetylated KGMH120 particles in the matrix tablet may form crosslinks between the interparticle surfaces through solid-state intermolecular interactions. When water was introduced, the particles slowly absorbed water from the tablet surface, forming a hydrogel between and inside the particles (Figure 10). Therefore, the disintegration of the tablet was suppressed (Figure 5), and the entire tablet became a stable gel matrix, which controlled drug release as a sustained-release tablet. In contrast, the KGMH0 matrix tablet might not form crosslinks between the interparticle surfaces; therefore, when water was introduced, the individual particles rapidly absorbed water (Figure 10), and the tablet disintegrated from the pressure of the expanded particles (Figure 5). This result also suggests that the disintegration time of matrix tablets can be changed to freely control the drug release rate by varying the degree of grinding.

## 4. Conclusions

Optimal formulations of KGM containing 16 *w/w*% Ca(OH)_2_ were prepared to develop a sustained drug release gel matrix tablet using a dry solid-state method without degrading the active ingredient of the drug, which occurs during wet granulation in the conventional method. Fine Ca(OH)_2_ particles were uniformly dispersed in KGM by mechanochemical treatment, and the deacetylation reaction of KGM with fine Ca(OH)_2_ particles in an alkaline sol may be accelerated by mechanochemical energy during solid grinding. It can be concluded that the compressed matrix tablets containing partially deacetylated KGM promoted the formation of strong gels, suppressed tablet disintegration, and controlled drug release following the Higuchi theory during the drug release test. These results indicate that the mechanochemical effect of KGM with Ca(OH)_2_ can freely control the drug release mechanism and its rate by changing the water penetration rate and disintegration time of KGM matrix tablets.

## 5. Materials and Methods

### 5.1. Materials

Theophylline anhydrate (TH) bulk powder was obtained from Shizuoka Caffeine Co., Ltd. (Lot No. F22224, Shizuoka, Japan). Water-soluble dietary fiber (KGM) was obtained from Shimizu Chemical Co., Ltd. (Leorex^®®^ RS, Mihara, Japan) as a food additive. Leorex^®®^ RS (KGM) is a polysaccharide with β-1 binding between D-glucose and D-mannose (ratio of approximately 1:1.6). Its molecular weight was approximately 1 million or more, its degree of polymerization was approximately 6200, and its molecular length was R0 = 1.3 Å. The viscosity of the 1.0 *w/v*% aqueous solution was 36,900 mPa·s at 25 °C, as previously reported [29]. Calcium hydroxide (Ca(OH)_2_) was obtained from Wako Pure Chemical Industries, Ltd. (Tokyo, Japan).

### 5.2. Grinding Treatment

The KGM formulation samples (KGML, KGMM, and KGMH) contained 5.2, 10.5, and 16.0 *w/w*% Ca(OH)_2_, respectively. Ten grams of the weakly mixed KGM and Ca(OH)_2_ powder samples were placed in a 250 mL agate ball mill pot containing 10 agate balls (∅15 mm × 10 mm) and were ground for 120 min at 200 rpm with a planetary ball mill crusher (P-6; Fritsch), respectively. The ground samples were labeled KGML120, KGMM120, and KGMH120. KGMH samples containing 16.0 *w/w*% Ca(OH)_2_ were ground for 0, 10, 30, 60, and 120 min and labeled KGMH0, KGMH10, KGMH30, KGMH60, and KGMH120, respectively. The temperature at the outside of the ball mill under the almost same experimental conditions was about 5 °C higher than room temperature, as reported previously [32].

### 5.3. Scanning Electron Microscopy with Energy-Dispersive X-ray Spectroscopy

The morphologies of the surface and the interior of the adsorbent particles were evaluated by scanning electron microscopy (SEM) (JSM-7600F, Field Emission SEM, JEOL, Ltd., Tokyo, Japan) with an accelerating voltage of 10 kV and 250× to 1000× magnification. Elemental analysis was performed using energy-dispersive X-ray spectroscopy (EDX) (JED-2300, JEOL, Ltd., Tokyo, Japan) at an accelerating voltage of 8.0 kV.

### 5.4. Powder X-ray Diffraction (XRD)

Powder X-ray diffraction (XRD) profiles of the ground samples were obtained using a RINT 2100 Ultima III (Rigaku Co. Ltd., Tokyo, Japan). The measurements were performed at 2°/min using an X-ray tube with Cu Kα radiation at a voltage of 40 kV and a current of 40 mA.

### 5.5. Fourier-Transform Infrared (FT-IR) Spectroscopy

Fifty milligrams of the sample powder was softly ground and dispersed in 950 mg of KBr powder in an agate mortar with a pestle. The FT-IR spectra of the sample powders were measured with 200 scans of powder diffuse reflectance on an FT-IR spectrophotometer (FT-IR/4100, Jasco Co., Ltd., Tokyo, Japan) and corrected using the Kubelka–Munk equation.

### 5.6. Tablet Preparation

Ground KGM powder (9.5 g) containing various amounts of Ca(OH)_2_ was placed with TH powder (0.5 g) in a plastic bag (1.0 L capacity) and mixed well by hand for 5 min. Then, 200 mg of the mixed KGM powder with 5 *w/w*% TH was placed in an 8 mm diameter die and pressed at 10.0 kN for 10 s with a flat-surface punch at a compression speed of 10 mm/min at about 25 °C using a compression/tension tester (Techno Graph TG-50kN, NMB Co., Ltd., Tokyo, Japan).

### 5.7. Drug Release Kinetics Measured by the Dissolution Test

Dissolution tests of the test tablets were performed in 900 mL of water in a 1000 mL capacity round-bottomed flask at 37.0 ± 0.5 °C with stirring at 100 rpm using a puddle in an automatic dissolution test apparatus (Type DT-610, JASCO Co., Ltd., Tokyo, Japan; Japanese Pharmacopeia 18). Dissolved drug concentrations were automatically measured by ultraviolet (UV) absorption at 271 nm at predetermined time intervals in quartz flow cells with a length of 10 mm using a UV–visible spectrophotometer [33]. All measurements were repeated thrice.

### 5.8. Water Penetration into Tablets

The rate of water penetration into the tablets was measured using a custom-modified glass pipette, as reported by Takayama et al. [34]. After placing the tablets on the glass filter of the modified glass pipette filled with water, the amount of water absorbed was measured at predetermined times.

## Data Availability

Not applicable.
